# A new prediction model for prognosis of patients with intermediate-stage HCC after conventional transarterial chemoembolization: an internally validated study

**DOI:** 10.7150/jca.34064

**Published:** 2019-10-21

**Authors:** Rong-Xin Chen, Yu-Hong Gan, Ning-Ling Ge, Yi Chen, Min Ma, Bo-Heng Zhang, Yan-Hong Wang, Sheng-Long Ye, Jian-Feng Luo, Zheng-Gang Ren

**Affiliations:** 1 Liver Cancer Institute, Zhongshan Hospital, Fudan University and Key Laboratory of Carcinogenesis and Cancer Invasion, Ministry of Education, Shanghai, China.; 2 Department of Health Statistics and Social Medicine, School of Public Health, Fudan University, Shanghai, China.

**Keywords:** Carcinoma, hepatocellular, Chemoembolization, therapeutic, Prognosis

## Abstract

**Purpose**: The prognosis of patients with intermediate-stage hepatocellular carcinoma (HCC) treated by conventional TACE (cTACE) is greatly heterogeneous. This study aimed to develop a new survival prediction model to help select patients who would benefit better from cTACE treatment.

**Methods**: We collected data of 848 treatment-naïve patients with BCLC B HCC who received cTACE as first-line therapy. The prognostic model's variables were derived from univariate and multivariate Cox regression analyses. The concordance index (C-statistic) calculated through cross-validation and bootstrap resampling was used for the model selection. The calibration of our final prediction model was also assessed.

**Results**: The model showed a better discrimination ability than Bolondi's BCLC B1-B4 subclassification to predict the prognosis of BCLC B patients (C-statistic, 0.66 *vs*. 0.60; difference, 0.05, 95% CI, 0.03-0.07). In cross-validation, bootstrap resampling demonstrated that the model maintained sufficiently discriminant (an average of C-statistic, 0.66; 95% CI, 0.65-0.68). The model calibration was accurate in predicting survival of patients matched well with the observed outcomes. On the basis of the improved survival of 18 months or more as the responding patient, the observations of patients in each response category (responder and non-responder) were fair-moderately matched with those predicted by the model (κ=0.40, *P*<0.001).

**Conclusions**: Based on clinically available features of patient, tumor and liver function, we developed an alternative prediction model with better performance than the Bolondi's substaging system for intermediate HCC patients after cTACE, which could help define the distinct subgroup of BCLC B patients who are suitable for cTACE treatment.

## Introduction

Hepatocellular carcinoma (HCC) is the sixth most common cancer and the third most common cause of cancer-related death worldwide 1. Most patients are diagnosed at the intermediate-advanced stage in their initial visit and unamenable to curative therapies. Conventional transarterial chemoembolization (cTACE) is the standard of care for the patients with intermediate-stage HCC 2. However, intermediate HCC patients are comprised of a highly heterogeneous population characterized from large to multiple lesions and different aspects of hepatic function, resulting that predicting the prognosis of those patients is a major challenge 3, 4. Nevertheless, accurate assessment of the individualized prognosis will instruct patient selection and treatment planning, as well as facilitate the effective doctor- patient communication.

Barcelona Clinic Liver Cancer (BCLC) staging system linking treatment indications to prognostic information has been proven instrumental in guiding treatment 2. However, optimal treatment for intermediate-stage (BCLC B) HCC patients is still a matter of heated debate because of considerable heterogeneous conditions of the patients, differences in the treatment modality between Western and Asian countries, and the disease etiology 5. Although two randomized clinical trials demonstrated a clear survival benefit of cTACE over best supportive care for BCLC B patients as a whole, not all such patients will derive similar benefit from cTACE and the overall outcomes are not satisfactory (2-year survival rates: 63% and 31%, respectively) 6, 7. This status triggers intense investigation of combined treatment or more aggressive treatments for BCLC B patients 8. The improved efficacy of adding radiofrequency ablation (RFA) to TACE for intermediate HCC has been increasingly reported 9, 10. Emerging data also showed that the survival advantage of hepatic resection over TACE was observed in the selected BCLC B patients 11-13. In Hong Kong Liver Cancer (HKLC) Staging System, survival benefit of resection is substantial in the subsets of BCLC B patients when compared with TACE 14. Bolondi et al 15 have proposed that BCLC B patients could be grouped into B1-B4 substages to tailor therapeutic algorithms. According this subclassification, TACE is an appropriate and first-line treatment option for BCLC B1, B2 patients. In contrast, Nouso et al 9 have reported that in those subgroup patients, RFA monotherapy is more effective than TACE. Moreover, this substaging system did not fully account for differences in the prognosis between the patients with HBV- and HCV-related HCC. Following Bolondi's BCLC B1-B4 subclassification, Kinki criteria, up-to-seven criteria with serum tumor markers, and combining tumor number, size with Child-Pugh grade were proposed to subgroup BCLC B patients for better predicting prognosis and making treatment decision 16-18. Anyway, it seems that various treatment strategies lead to different outcomes in BCLC B patients. In a real-world practice setting, some patients progress despite TACE treatments and have a poor prognosis. A new model to reflect how clinical factors determine the prognosis of BCLC B patients who underwent cTACE treatment and identify which population of BCLC B patients might benefit from cTACE is needed. This will also reduce unnecessary TACE treatments for those patients who will not benefit and allows them to receive alternative treatment modalities.

In this study, we developed a new prediction model for prognosis of intermediate-stage HCC patients treated by cTACE on the basis of a large cohort from a single tertiary referral center, which may be used in evaluating the individualized prognosis and helping to select the patients appropriate for cTACE treatment.

## Materials and Methods

### Patients

This retrospective study was approved by the Institutional Review Board of our Hospital. Informed consent was waived because of the retrospective nature of the study. The inclusion criteria of this study consisted of 1) HCC diagnosis by liver biopsy or typical radiologic appearance; 2) treatment-naive HCC patients who received TACE as first-line therapy; 3) intermediate-stage HCC (BCLC stage B according to BCLC staging system 2018 version or stage Ib, IIa, IIb judged by Guidelines for Diagnosis and Treatment of Primary Liver Cancer in China 2017 Edition) 19 : single tumor larger than 5 cm or 2-3 tumors larger than 3 cm, 4 or more tumors irrespective of size; no vascular invasion or extrahepatic metastasis; 4) preserved liver function of Child-Pugh grade A or B; 5) performance status of ECOG 0-2; 6) alanine or aspartate aminotransferase levels of less than five times the upper normal limit; 7) serum creatinine level of less than 177 µmol/L. The exclusion criteria were: 1) patients underwent surgical resection or liver transplantation for HCC after cTACE; 2) diffuse type of HCC; 3) ruptured HCC; 4) prior or current other malignancies. In accordance with these criteria, the final study population included 848 patients who received TACE as first-line treatment during 2007 to 2014 at the department of hepatic oncology, Zhongshan hospital of Fudan University, Shanghai, China. From our prospectively maintained database, we retrieved the variables related to patients' demographics, clinical features, laboratory results, tumor characteristics, treatment course and survival data of each patient.

### Development of the prognostic model

The variables selected for the prediction model were derived from univariate and multivariate analyses of patient, tumor, liver function and treatment-related factors using a Cox proportional hazards regression. The output of the model for estimating the survival probability was presented as coefficients which were used to calculate hazard ratios: S(t)=S_0_(t) ^exp(∑Xβ)^ where X is the vector of independent variables, X=(x_1_, x_2_,…x_k_), and β is the vector of the regression coefficient for the corresponding variables. S(t) is the probability of surviving past time t and S_0_ is the baseline survival probability.

### Performance validation

C-statistic (concordance index) is defined as the probability of concordance between predicted probability and outcome. In cross-validation, the bootstrap was applied by dividing the derivation data into the training and validation subsets at the ratio of 7:3 in sample size. C-statistic was calculated through the methods of cross-validation and bootstrap (1,000 data re-samplings) to assess the discriminatory ability for model selection. Then, calibration was assessed by comparing the mean model-predicted survival probabilities of the groups with the observed survival. The observed outcome was plotted against the model-predicted survival to create a calibration plot. Bolondi's BCLC B1-B4 subclassification 15 and Hong Kong Liver Cancer (HKLC) staging system 14 were applied to our patient population and its discriminatory power was compared with that of our model.

### cTACE therapy

All procedures of lipiodol TACE were performed according to our institutional protocol as previously described 20-22. Initially, multiple angiographies were performed to identify hepatic artery anatomy, tumor staining and the tumor-feeding artery. The 5F RH catheter (Cook Co., USA) was advanced into the desired hepatic artery as close as possible to the tumor for selective (lobar/segmental) injections. Microcatheters (2.7 Fr Progreat Microcatheter, Terumo Co., Tokyo, Japan) were used to catheterize the feeding artery if needed for superselective (subsegmental) injection. Chemotherapeutic agents such as 5-fluorouracil 1.0 g, cisplatin 80mg or oxaliplatin 150 mg were slowly infused followed by an infusion of mitomycin C 10-20 mg or epirubicin 30-50mg and Lipiodol 5-20 ml emulsion. The regimen was adjusted depending on liver function, peripheral leukocyte and platelet levels. When embolization with lipiodol mixture was insufficient, additional embolization with gelatin sponge particles were performed.

cTACE was repeated on an “on-demand” basis at the interval of 6-8 weeks in the presence of residual viable tumor or recurrence on follow-up imaging. Combined with other locoregional treatment (including ethanol injection, radiofrequency ablation, microwave, external beam radiation), or sorafenib was allowed to treat residual tumor or tumor progression after cTACE according to tumor size, number, location, liver function and particular purposes. Complications were classified according to the Society of Interventional Radiology (SIR) guidelines 23.

### Statistical analysis

Baseline data are presented as median values with interquartile range for quantitative variables and percentages for categorical variables. The primary end point in this analysis was the patient's overall survival, which was calculated from the date of first TACE to the date of death from any cause, the date of the last follow-up or the date of data censoring (March 31, 2015). Survival curves were estimated using Kaplan-Meier analysis and significant differences were determined with log-rank tests. The univariate and multivariate Cox proportional hazards model was performed to identify the independent predictors of survival. The C-index values was used to compare the discriminatory abilities of the models for predicting survival. The degree of interobserver agreement was calculated using kappa (κ) testing. A κ value of 0.6-0.8 or more than 0.8 was considered excellent or almost perfect agreement, 0.4-0.6 good agreement, and less than 0.4 poor agreement. All statistical analyses were conducted with SAS software (version 9.2, SAS institute Inc., Cary, NC). A *P* value of < 0.05 was considered statistically significant.

## Results

### Patient profile

There were 848 intermediate HCC patients included in this study. The characteristics of the study population are presented in Table 1. Overall, the median age was 58 years. Most of patients were men (732, 86.3%) and were predominantly hepatitis B surface positive (680, 80.2%). Eight hundred eight (95.3%) patients had liver function of Child-pugh grade A, 40 (4.7%) had grade B liver function. Six hundred twenty-five (73.7%) patients had a tumor size greater than 5 cm, and 369 (43.5%) patients had a solitary tumor. One hundred twenty-six patients received combined treatments (89 ablation, 21 external radiation, 16 sorafenib) for treating residual tumor or tumor progression after cTACE.

**Figure 1:**
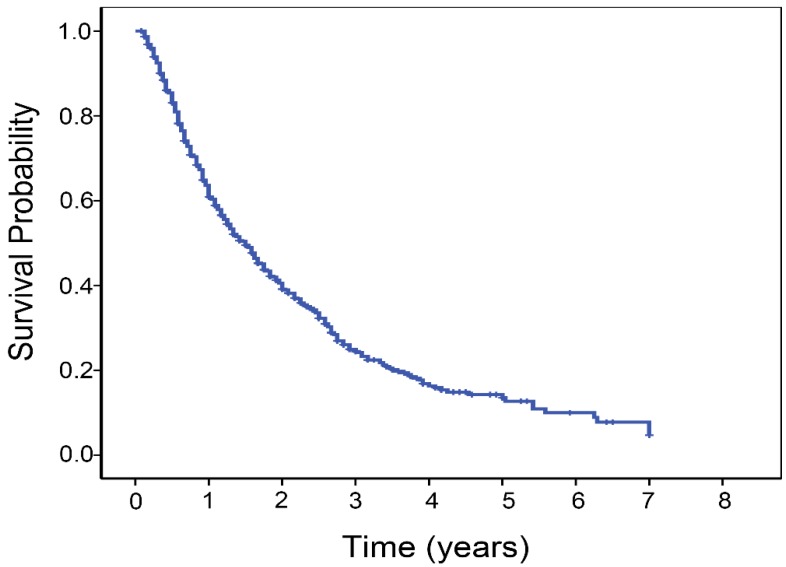
Kaplan-Meier estimated survival curves

The follow-up period ranged from 1.0 to 84.0 months, with a mean of 17.3 months. The median OS time was 18.0 months (95% confidence interval, 16.0-20.0 months). The 1-, 3-, and 5-year overall survival rates of patients were 60.9%, 24.4% and 13.5%, respectively (Fig. 1). A total of 541 patients (63.8%) died during the study period. The distribution of the cause of death consisted of cancer death or hepatic failure in 471 patients, rupture of esophago-gastric varices in 54, rupture of HCC in 8 and other causes in 8. In total, 848 patients underwent 2850 TACE sessions (median, 3 sessions, interquartile range 2-4 sessions per patient). Post-embolization syndrome (characterized by nausea, vomiting, fever, and abdominal pain) after cTACE was the common minor complication in most patients. There were sixteen major complications: three hepatic failure, three bleeding from ruptured esophago- gastric varices, three severe sepsis, one pulmonary embolism, one acute pancreatitis, two tumor rupture, and three femoral pseudoaneurysm.

### Prognostic prediction model

Variables in the prediction model were selected based on univariate and multivariate analysis using Cox proportional hazards model. Univariate analysis showed albumin, bilirubin, GGT, ALT, prothrombin time, AFP, tumor size of the largest nodule, and combined treatment to be statistically significant factors affecting survival. Child-Pugh grade as a composite factor was not included in the initial analysis. Despite the effect of gender and age was not significant in univarariate analyses, the two variables were judged a priori to be clinically sound. Multivariate analysis revealed that gender (P=0.033), age (P=0.037) (patient factors); albumin (*P* <0.001), bilirubin (*P*=0.003), GGT (*P* <0.001) (liver function); tumor size of the largest nodule (*P* <0.001) (tumor-specific relevance), combined treatment (*P* <0.001) (treatment-related factors) were the significant prognostic factors in the patients' prognosis (Table 2).

Based on the results of the Cox proportional hazards model, several prediction models were constructed to predict the patients' prognosis (Table 2). The initial model (Model 1) included six variables of gender, age, albumin, bilirubin, GGT, tumor size of the largest nodule. The variable of combined treatment was felt to be different from other assessed pre-therapy clinical factors, which was not included in the initial model. To further improve model efficiency with as fewer numbers of variables as possible, the model was optimized through a stepwise procedure. The C-statistic analysis through cross-validation and bootstrap resampling was calculated to appraise the predictive ability of the model. With the stepwise addition of AFP, tumor number and combined treatment information, there was a small increase of C-statistic that was very close to the performance of the original model (Table 3). Therefore, the model 1 was selected as the final prediction model (C-statistic of 0.66). A concrete calculation formula for estimating survival probability was expressed as follows: S(t)=S0(t)^exp(∑Xβ)^, ∑Xβ=0.005*age-0.269*gender-0.383*albumin+0.009*bilirubin+0.001*GGT+0.086* (tumor size). Because the selected model included the available pre-therapy clinical factors, it could also be used as the “pre-treatment survival prediction model” which would help predict the patients who would benefit from cTACE before treatment.

### Validation

**Figure 2:**
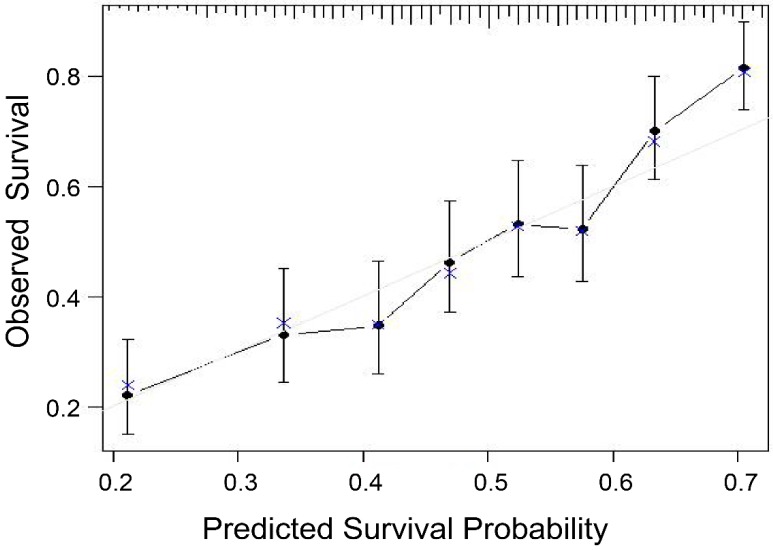
Calibration curve of the model in patient cohorts. The model seems to yield accurate survival prediction.

After the new prognostic model was developed, its performance was assessed. Using the bootstrap method, the model maintained sufficiently discriminatory performance (C-statistic of 0.66, 95% CI, 0.65-0.68). A calibration chart between predicted and observed survival was plotted. The calibration of the survival prediction model yielded accurate predictions of survival in cTACE-treated BCLC B patients (Fig. 2).

### Prediction ability of the model *versus* BCLC B1-B4 subclassification

When compared the performances of Bolondi's BCLC B1-B4 subclassification (the value of C-statistic, 0.60, 95% CI, 0.59-0.63), our survival prediction model had a better discriminatory ability for BCLC B patients treated by cTACE (C-statistic, 0.66 *vs*. 0.60, difference, 0.05, 95% CI, 0.03-0.07). Moreover, this new model also showed a better predictive performance than HKLC staging system (C-statistic, 0.59; 95%CI: 0.57-0.60) to predict the prognosis of intermediate-stage HCC patients who underwent cTACE treatment.

### Prognostic prediction model stratifying respondents and non-respondents

Using the cut-off of the improved survival of 18 months to stratify the patients as responders and non-responders to cTACE, the actual observations of patients in each response category (responder and non-responder) were fair-moderately matched with those predicted by the survival prediction model (the patient of 18-month survival probability > 0.5 was considered as the responder) (κ=0.40, *P* < 0.001) (Table 4).

### Role of the combined treatment after cTACE

**Figure 3:**
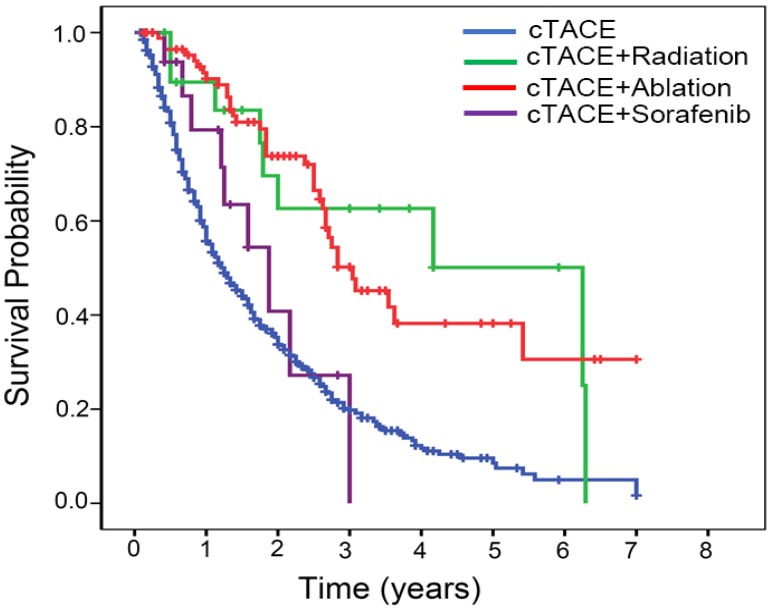
Kaplan-Meier estimated survival curves by the combined treatment modality.

We observed the superior survival rates in selected BCLC B patients who received the combined treatment (ablation or radiation) after cTACE (cTACE+ablation *versus* cTACE, *P*<0.001; cTACE+radiation *versus* cTACE, *P*=0.001) (Fig. 3), suggesting that combined treatment could contribute to prolong the survival of subgroup of intermediate-stage HCC patients.

## Discussion

The principal finding of this study is that an alternative prediction model with better performance than the Bolondi's substaging system is developed for estimating the survival of intermediate-stage HCC patients who underwent cTACE treatment. Furthermore, the new model has good performance in predicting survival of patients matched well with the observed outcomes and enables the stratification of responders versus non-responder to cTACE. Therefore, this model helps identify the distinct subgroup of patients who are suitable for cTACE treatment in the decision-making with regard to administration of a TACE treatment, yielding a better survival outcome in certain subgroup of intermediate-stage HCC patients. In addition, this model is mainly derived from the patients with HBV-related HCC, which would be more valuable for assessing the prognosis of intermediate HCC patients with HBV background who received cTACE treatment. So, our findings have clinical implications in predicting and improving the survival outcome of intermediate-stage HCC patients who underwent cTACE treatment.

The BCLC staging system shows broad survival variations for tumors of the same stage, especially not accounting for the heterogeneity of the intermediate patients 24-26. Although Bolondi's substaging system of BCLC B1-B4 easily stratifies patients, it does not always show the accuracy in predicting patient-specific survival 27, 28. Furthermore, this substaging system did not take into account the difference in prognosis between the patients with HBV-related and HCV-related HCC. Recently, a study 29 have showed that Bolondi's BCLC B1-B4 subclassification does not stratify perfectly patients treated with cTACE except substage B1 patients and some patients of substage B4 also can benefit from cTACE. To achieve a better method of predicting prognosis for intermediate HCC patients, various criteria and nomograms have been developed 16-18, 28, 30, 31. However, no accepted model has been proven capable of providing accurate predictions of prognosis in cTACE-treated intermediate HCC patients. Given the wide application of cTACE in real clinical practice (approximate 30-40% of entire HCC patients) 32, it is urgent to well know factors affecting treatment outcomes and establish an appropriate prognostic model which could be clinically valuable in guiding patient selection to maximize the benefits of cTACE. Our model showed a better discrimination ability than Bolondi's BCLC B1-B4 subclassification to predict the prognosis of BCLC B patients who underwent cTACE treatment. Furthermore, this prediction model underwent internally cross-validation and better predicted the long-term outcomes in our cohort. Using available objective clinical parameters and statistical methods, our survival prediction model accounts for the interaction of relevantly and statistically significant factors to provide accurate information for the prognosis in intermediate HCC patients treated by cTACE. Our prognostic model incorporates the indispensable factors for clinical management such as the patients' demographic characteristics, liver function and tumor parameters. To develop this model, we analyzed the age, albumin, bilirubin, GGT and tumor size treated as continuous variables rather than dichotomous or categorical variables because these factors could exhibit prognostic characteristics more ideally expressed by gradual increases, which captures subtle changes of clinical variables and adds the strength and the high discrimination power to our model. This is an important distinction from the previous nomograms and Bolondi's BCLC B1-B4 subclassification. Although many prevalent prediction systems use some of the variables as categorical or ordinal variables (e.g. Child-Pugh). However, for example, the Albumin-Bilirubin (ALBI) grade is entirely objective means to evaluate liver function in patients with HCC through a complex formula: (log 10 bilirubin × 0.66) + (albumin × -0.085). Serum albumin and bilirubin concentrations are analyzed as continuous variables. It has been reported that ALBI as a prognosticator is superior to Child-Pugh among HCC patients in some studies. Even more, ALBI is proposed as stratification factor within the Child-Pugh A class. Our prediction model includes the variables of albumin, bilirubin, GGT and tumor size treated as continuous variables. On the other hand, although Bolondi's BCLC B1-B4 subclassification has the advantage of the relative simplicity, it is limited by its inability to account for the interaction of various clinical characteristics, illustrating the difficulty of determining the individual patient's survival 27. Through integration of interactions among various prognostic factors and statistical tools, our prediction model showed a better discriminatory performance compared with Bolondi's subclassification to predict the prognosis in cTACE-treated intermediate HCC patients. In addition, our model incorporates objective parameters (e.g. albumin and bilirubin, instead of the Child-Pugh score).

The prediction model did not include the variable of combined treatment, albeit it affected the prognosis of patients. First, it could not significantly improve the model's predicative performance. Second, the need for combined treatment was mainly determined by baseline tumor size, number of tumor nodules, site, liver function and each physician's preference. These patients with better performance status and compensated liver disease are likely allocated to combined treatment. In contrast, those patients who performed poorly will not be candidates to combined therapy. Therefore, for this reason, this variable as a time-dependent covariate should be out of the model. Third, the variable of combined treatment after cTACE was different from other baseline clinical factors that could obtained before treatment.

In the present study, TACE combined with other treatment approach (ablation or radiation) contributed to prolong the survival of the selected BCLC B patients. However, the number of patients who received combined treatments was small and selection bias could not be ruled out. This preliminary result is consistent with the conclusion from the previous reports 33, 34. This needs to be validated by the randomized clinical trials in the future.

Our predication model integrated with the pre-treatment available clinical parameters aids in planning treatment strategies for intermediate-stage HCC patients. If a treatment-naïve intermediate HCC patient is stratified into responder predicted by our model, cTACE treatment is preferred. On the contrary, if the patient's survival is less than 18 months predicted by our model (judged as a non-responder to cTACE), the patient should avoid cTACE monotherapy or be assigned to combined or alternative treatments, or clinical trials.

There are several limitations in this study. First, this study was retrospective in nature, therefore, it was subject to potential bias. This current model was constructed only with basal variables. Other clinical and laboratory parameters that might have effects on prognosis of HCC patients are not included. For example, despite performance status is an inherently subjective variable, it is a well-known prognostic factor. The presence of portal hypertension (e.g. platelet count, prior ascites, varices) is an important predictive factor. However, our prediction model has the potential of incorporating additional patient-, tumor- and treatment-relevant variables into this model to strengthen its discrimination power. Second, the median survival of patients in our cohort was 18 months, which is close to the result from Lencioni R et al. (19.4 months) 35 but is significantly shorter than those of gained survival benefit in some previous reports 36, 37. This is associated with the fact that decompensated patients (4.7% were Child-Pugh class B) and the patients with ECOG PS 1-2 were recruited in our study. Third, the calculation in our model is rather complicated. However, with the availability of smartphone App and website application, doctors can easily use this model to conduct the bedside calculation. Since artificial intelligence based on machine learning predicts tumor response to TACE in patients with HCC 38, it will also be useful to determine the prognosis of patients receiving TACE treatment and select which patients benefit from TACE treatment. Fourth, we only performed an internal validation. However, the predictive performance of this model needs external validation and multicenter prospective cohort validation before it can be used in clinical practice.

In conclusion, we developed a new prediction model with a better performance for predicting the prognosis of intermediate-stage HCC patients who underwent cTACE, which may be helpful in selecting patients suitable for cTACE treatment to yield a better survival outcome, especially in the patients with HBV background.

## Figures and Tables

**Table 1:** Baseline characteristics of the study patients

Variables	All patients (N=848)
Age (y)*	58 (49-65)
Gender (male/female)	732/116
HBsAg (positive/negative)	680/168
HCV antibody (positive/negative)	24/824
Albumin (g/L)*	37(34-41 )
Bilirubin (umol/L)*	12.2 (8.8-17.4)
GGT (IU/L)*	123.0 (71.3-217.8)
ALT (IU/L)*	40.5 (26.0-63.0)
Prothrombin time (s)*	12.7 (11.9-13.7)
AFP (ng/mL) *	264.0 (12.0-5359.5)
Child-Pugh grade (A/B)	808/40
Tumor size, diameter of largest tumor (cm)*	8.0 (5.0-10.0)
Tumor number (1/2/3/>3)	369/169/98/212
TACE sessions*	3(2-4)
Combined treatment (PEI, RFA, microwave/ external radiotherapy/sorafenib)	89/21/16
BCLC B subclassification (B1/B2/B3+B4)	181/468/199
Survival times, median (95% CI)	18.0 (16.0-20.0)

*Values are medians, with interquartile ranges shown in parentheses. HbsAg: hepatitis B virus surface antigen; HCV: hepatitis C virus; BCLC: Barcelona Clinic Liver Cancer; AFP: α-fetoprotein; GGT: γ-glutamyltranspeptidase; ALT: alanine aminotransferase.

**Table 2:** Variables selected for the prediction model: univariate and multivariate analyses of the variables associated with survival of BCLC B patients after cTACE treatment

Variable	Univariate		Multivariate	
	Hazard Ratio (95% CI)	*P* value	Hazard Ratio (95% CI)	*P* value
Age	0.999 (0.992-1.006)	0.710	1.008 (1.000-1.015)	0.037
Gender	0.847 (0.671-1.069)	0.162	0.773 (0.611-0.979)	0.033
HBsAg	0.991 (0.805-1.220)	0.930		
HCV antibody	1.028 (0.642-1.645)	0.909		
Albumin	0.629 (0.528-0.749)	<0.001	0.714 (0.595-0.856)	<0.001
Bilirubin	1.009 (1.004-1.015)	0.001	1.009 (1.003-1.015)	0.003
GGT	1.001 (1.001-1.002)	<0.001	1.001 (1.000-1.001)	<0.001
ALT	1.002 (1.001-1.003)	0.004		0.242
Prothrombin time	1.046 (1.007-1.087)	0.019		0.135
AFP	1.000 (1.000-1.000)	<0.001		0.087
Tumor size	1.096 (1.071-1.121)	<0.001	1.086 (1.061-1.112)	<0.001
Tumor number	0.967 (0.902-1.036)	0.339		
Combined treatment	0.628 (0.544-0.725)	<0.001	0.667 (0.577-0.771)	<0.001

HbsAg: hepatitis B virus surface antigen; HCV: hepatitis C virus; AFP: α-fetoprotein; GGT: γ-glutamyltranspeptidase; ALT: alanine aminotransferase.

**Table 3:** Comparing the prediction ability of different models using the C-statistic

Model	Variables	C-statistic (95% CI)
1	Age, Gender, Albumin, Bilirubin, GGT, Tumor size	0.66 (0.65~0.68)
2	Model 1, AFP	0.65 (0.65~0.68)
3	Model 1, AFP, Tumor number	0.67 (0.65~0.69)
4	Mode 1, AFP, Tumor number, Combined treatment	0.69 (0.67~0.71)

AFP: α-fetoprotein; GGT: γ-glutamyltranspeptidase.

**Table 4:** The patients as the observed responders and non-responders compared with those predicted by the model

Observed category	Predicted category		Total
	Responder	Non-responder	
Responder	286	97	383
Non-responder	158	307	465
Total	444	404	848

Note: the patient alive at the final data censoring and follow-up time less than 18 months was regarded as the responder.

## Abbreviations

HCC: hepatocellular carcinoma
cTACE: conventional transarterial chemoembolization
c-statistic: concordance index
CI: confidence interval
BCLC: Barcelona Clinic Liver Cancer
RFA: adiofrequency ablation
AFP: α-fetoprotein
GGT: γ-glutamyltranspeptidase
ALT: alanine aminotransferase
HbsAg: hepatitis B virus surface antigen
HCV: hepatitis C virus
